# Prevalence and impact of sleep disorders in patients with hereditary angioedema: A multicenter cross-sectional study in Latin America

**DOI:** 10.1007/s11325-026-03815-8

**Published:** 2026-09-25

**Authors:** Ivan Cherrez-Ojeda, Thomas Rott, Karla Robles-Velasco, Ileana María Madrigal Beas, Sandra Nieto-Martinez, María Margarita Olivares, Gonzalo Chorzepa, Oscar Calderon, Juan José Matta Campos, Blanca Maria Morfin-Maciel, Guillermo Guidos, German Dario Ramón, Dario Josviack, Nora Alarcon Cedeño, Gabriela Rodas-Valero, Marco Faytong-Haro, Jonathan A. Bernstein, Markus Magerl, Henriette Farkas, Leonie Herzog, Thomas Buttgereit

**Affiliations:** 1 Charité – University Medical Center Berlin, a corporate member of Freie Universität Berlin and Humboldt-Universität zu Berlin, Institute of Allergology, Berlin, Germany; 2 Fraunhofer Institute for Translational Medicine and Pharmacology ITMP, Immunology and Allergology, Berlin, Germany; 3 Universidad Espíritu Santo, Samborondón, Ecuador; 4 Respiralab Research Group, Guayaquil, Ecuador; 5 Henry Ford Sleep Center, 2799 West Grand Boulevard, CFP-3, Detroit, MI 48202 USA; 6 Associate professor of the speciality of Allergy and Clinical Immunology High Specialt Medical Unit, Specialty Hospital of the National Medical Center of the West IMSS., Guadalajara, Jalisco México; 7 Instituto Nacional de Pediatría. Unidad de Genética de la Nutrición, Ciudad de México, Mexico; 8 Clínica Medellín Poblado; Grupo de Errores Innatos de la inmunidad-GEII-(IDPs) y Centro Nacional “Jeffrey Modell”, Universidad de Antioquia, Medellín, Colombia; 9 Sanatorio Parque, Rosario, Argentina; 10 ACARE Centre, Clinica SANNA El Golf, Lima, Peru; 11 Médico Pediatría y Alergia. Consulta privada, Mexico city, México; 12 Hospital San Angel Inn Chapultepec, Ciudad de Mexico, Mexico; 13 S.E.P.I—E.N.M.H Instituto Politecnico Nacional, Mexico city, Mexico; 14 Hospital Italiano Regional del Sur, Bahia Blanca, Argentina; 15 Instituto de Medicina Respiratoria, Rafaela, Santa Fe Argentina; 16 Hospital General Portoviejo—IESS, Portoviejo, Ecuador; 17 Universidad Estatal de Milagro, Cdla. Universitaria, “Dr. Rómulo Minchala Murillo” – km. 1.5 vía Milagro – Virgen de Fátima, Milagro, Guayas 091050 Ecuador; 18 Ecuadorian Development Research Lab, Daule, Guayas Ecuador; 19 Sociology and Demography Department, The Pennsylvania State University, University Park, PA USA; 20 Division of Rheumatology, Allergy and Immunology, Department of Internal Medicine, University of Cincinnati College of Medicine, Partner Bernstein Allergy Group and Bernstein Clinical Research Center, Cincinnati, USA; 21 Hungarian Angioedema Center of Reference and Excellence, Department of Internal Medicine and Haematology, Semmelweis University, Budapest, Hungary

**Keywords:** Hereditary angioedema, Insomnia, Severity, Sleep apnea, Sleep disorders, Quality of life

## Abstract

**Background:**

Hereditary angioedema (HAE) is a rare disease characterized by recurrent swelling episodes that may affect sleep and quality of life. Because data on sleep disorders in Latin American patients with HAE are limited, we assessed their prevalence and clinical correlates using structured sleep and HAE patient-reported outcome measures.

**Objective:**

To assess the prevalence/impact of sleep disorders in HAE patients using validated tools.

**Methods:**

This cross-sectional, multicenter online survey included adult patients (> = 18 years) with clinician-confirmed HAE from 13 reference centers in five Latin American countries. Data were collected between August and December 2023. Sleep was assessed using the Global Sleep Assessment Questionnaire (GSAQ), SATED scale, and STOP-Bang; HAE was assessed using the Hereditary Angioedema Activity Score (HAE-AS), Angioedema Control Test (AECT), and Angioedema Quality of Life questionnaire (AE-QoL). Logistic regression models were adjusted for age, sex, and BMI.

**Results:**

The final analytic sample included 139 participants. Most patients had HAE-C1INH type 1 (64.0%) and were female (68.4%). Only 6.5% had good sleep health, and the mean SATED score was 5.46 (1.87). Insomnia was reported by 54.0% of participants, and 34.5% had STOP-Bang moderate-to-high OSA risk. In age-, sex-, and BMI-adjusted models, severe HAE activity was associated with higher odds of insomnia (OR = 2.62, 95% CI: 1.28–5.35, P = 0.008), RLS/PLMD (OR = 5.85, 95% CI: 1.59–21.62, P = 0.008), and STOP-Bang moderate-to-high OSA risk (OR = 2.37, 95% CI: 1.01–5.55, P = 0.047). Higher AE-QoL fatigue/mood and fears/shame scores were associated with several sleep outcomes. AECT control status did not show a consistent independent association after simultaneous adjustment.

**Conclusion:**

In this exploratory cross-sectional study, HAE activity and quality-of-life impairment were associated with several sleep outcomes. Targeted sleep screening may be useful in HAE care pathways if these associations are confirmed in prospective studies.

**Supplementary Information:**

The online version contains supplementary material available at https://doi.org/10.1007/s11325-026-03815-8.

## Introduction

Hereditary angioedema (HAE) is a rare genetic condition with an estimated global prevalence of 1:50,000 [1]. The condition results from either a deficiency or malfunction of the C1 inhibitor (C1INH), which disrupts the regulation of bradykinin production [2–6]. HAE-C1INH-Type 1 is the most prevalent, marked by insufficient levels of C1INH. HAE-C1INH-Type 2 is defined by dysfunctional C1INH. HAE due to FXII mutations (HAE-FXII) belongs to the even more rare subtype of HAE, i.e. HAE with normal C1INH (HAE-nC1INH) and has been shown to be estrogen dependent [7]. Despite the differences in their underlying mechanisms, all three types present similarly, with recurrent and unpredictable swelling episodes affecting the subcutaneous and submucosal tissues [6, 8]. These episodes can become life-threatening, particularly when involving the upper airway [9]. Angioedema is the third most common skin condition in emergency medicine. Additionally, HAE can significantly impair daily functioning, diminish quality of life (QoL), and burden affected individuals, their families, and society [10–14].

Sleep is a natural, reversible condition crucial for physiological functions in the body; non-REM (rapid eye movement) and REM sleep facilitate memory consolidation and the processing of sensory information. Moreover, quality sleep is essential for numerous physiological activities, including immunity, cardiovascular regulation, endothelial performance, hormone responses, brain circuitry, and the equilibrium between sympathetic and parasympathetic systems, ultimately resulting in optimal health [15–19] and QoL [20, 21] Impaired sleep affects a broad spectrum of physiological and psychological domains, including increased inflammation and [22] associated symptoms, mental and physical fatigue, mood disorders, excessive daytime sleepiness, and a significant reduction in overall QoL [23]. Longitudinal and experimental studies have associated chronic sleep deprivation or impairment with a range of severe comorbidities, including psychiatric and neurological disorders, diabetes, hypertension, and heart attacks [24–38], impaired glycemic control in diabetes [39, 40], and large-scale data show a U-shaped association between OSA and all-cause mortality [41], while the prognostic significance of daytime sleepiness varies by age [42]. The impact of impaired sleep on inflammatory skin conditions has been studied in chronic dermatoses, including atopic dermatitis (AD) [43–52] and chronic urticaria [53–55]. Similar sleep disturbance has also been reported in other chronic autoimmune conditions such as systemic sclerosis [56]. Christiansen et al. indicated that 35% of HAE-C1INH patients experienced sleep disorders without detailing the exact sleep condition. Banerji et al. identified sleep apnea (10%) as a self-reported comorbidity in patients with HAE.

Studies have also found that higher disease activity, poor chronic urticaria control, and impaired QoL come with a significantly increased risk for obstructive sleep apnea (OSA) [57]. Considering the limited evidence and the significance of sleep disorders in relation to inflammatory diseases, we intend to investigate the prevalence and impact of sleep disorders in patients with HAE using validated assessment tools and identify predictors associated to specific sleep disorders.

## Methods

### Study design

This was an observational, cross-sectional, multicenter, online survey of patients over ≥ 18 years of age diagnosed with HAE. Data collection took place between August and December 2023across 13 specialized HAE reference centers in five Latin American countries: Mexico (n = 5), Argentina (n = 3), Peru (n = 2), Colombia (n = 1), and Ecuador (n = 2).

### Participants and eligibility

The sampling frame consisted of patients currently under clinical follow-up at the participating reference centers. Inclusion criteria required participants to be ≥ 18 years of age with a diagnosis of HAE confirmed by laboratory parameters prior to enrollment. Exclusion criteria included a diagnosis of acquired angioedema, insufficient literacy to complete the survey independently, lack of informed consent, or incomplete data regarding critical study variables.

### Data collection and survey flow

The survey was hosted on the QuestionPro® platform and utilized a bifurcated data collection strategy to ensure high diagnostic accuracy. Unique access links were distributed to both the treating physicians and their respective patients. Physicians provided objective clinical data for each participant, while patients completed the Patient-Reported Outcome Measures (PROMs).

The survey followed a rigorous attrition flow: of the 1,070 landing page views, 635 responses were initiated, and 300 were completed (comprising 150 paired physician and 150 patient forms). After unifying these entries into single patient records and cleaning for missing data on critical variables, a final sample of 139 patients was included in the analysis.

Because the analytic dataset was anonymized, center identifiers were not retained after physician–patient record linkage. Therefore, center-level mixed-effects models and center-clustered robust standard errors could not be estimated. Country-level clustering was also not modeled because only five countries contributed participants and some country strata were small, making cluster-robust estimates unstable.

### Sample size and representativeness

Because HAE is a rare disease and the study used a multicenter convenience sampling frame, no a priori power calculation was performed for individual regression coefficients. The sample size was determined by the number of eligible participants who completed paired physician–patient forms during the study period. Based on the estimated HAE population across the five participating countries, the final sample represented approximately 4.1% of the theoretical regional HAE population. Regression analyses were therefore treated as exploratory and hypothesis-generating.

### Variables and instruments

The survey gathered demographic and clinical data. Objective clinical variables, including HAE diagnosis, subtype, medications, and comorbidities, were provided by treating physicians; PROMs, sleep measures, triggers, and other patient-reported variables were completed by patients.

All PROMs were administered in Spanish. Validated Spanish versions were used when available: the Mexican Spanish version of the AE-QoL and the Argentinian Spanish version of the AECT were employed. Validated Spanish versions were not available across all participating countries for the HAE-AS, GSAQ, and SATED; therefore, ad hoc Spanish translations were used for these instruments.

#### Disease-specific measures

HAE activity was assessed with the HAE-AS, with scores > = 12 classified as severe activity. Disease control was assessed with the AECT, with scores > = 10 classified as controlled disease and scores < 10 classified as uncontrolled disease. AE-QoL domain and total scores range from 0 to 100, with higher scores indicating greater quality-of-life impairment [58–60].

#### Sleep assessment measures

The GSAQ was used to screen for insomnia, GSAQ-defined sleep apnea symptoms, hypersomnolence, RLS/PLMD, REM-SBD, parasomnias, and SWSD. STOP-Bang [61–63] scores were categorized as low risk (0–2), intermediate risk (3–4), and high risk (5–8); the primary analysis used a score > = 3 to define moderate-to-high OSA risk. SATED scores range from 0 to 10, with higher scores indicating better sleep health; for descriptive purposes, scores were categorized as poor sleep health (0–5), intermediate sleep health (6–8), and good sleep health (9–10). COMISA was defined as concurrent GSAQ-defined insomnia and STOP-Bang moderate-to-high OSA risk in the same participant [64].

### Statistical analysis

Data were analyzed using Stata 18.0 (StataCorp LLC, College Station, TX, USA). The analytic sample included participants with complete data for age, sex, BMI, neck circumference, HAE-AS, AECT, AE-QoL domain scores, GSAQ, SATED, and STOP-Bang. Implausible BMI values were excluded during data cleaning, and no imputation was performed. Continuous variables are reported as mean (SD), and categorical variables are reported as n (%). Percentages were calculated using the final analytic denominator (N = 139) unless a smaller denominator is explicitly stated.

Primary binary sleep outcomes were GSAQ-defined insomnia, GSAQ-defined sleep apnea symptoms, hypersomnolence, SWSD, RLS/PLMD, REM-SBD, parasomnia, and STOP-Bang moderate-to-high OSA risk. STOP-Bang was analyzed primarily as low risk (0–2) versus moderate-to-high risk (3–8) and secondarily using the standard low (0–2), intermediate (3–4), and high (5–8) risk strata. SATED was described using the prespecified categories of poor sleep health (0–5), intermediate sleep health (6–8), and good sleep health (9–10), and was analyzed continuously as a 0–10 score in sensitivity analyses. COMISA was defined as concurrent GSAQ-defined insomnia and STOP-Bang moderate-to-high OSA risk in the same participant.

Logistic regression models were used for binary sleep outcomes. All sleep-outcome models were adjusted a priori for age, sex, and BMI. Clinical models additionally included the exposure of interest, namely severe HAE activity, AECT control status, or AE-QoL domain score. To limit overfitting in this rare-disease sample, clinical predictors were modeled separately in the primary adjusted models. Exploratory full models including HAE activity, AECT control status, AE-QoL domains, age, sex, and BMI were used to evaluate whether AECT retained an independent association after simultaneous adjustment. Standard STOP-Bang strata were evaluated using ordinal logistic regression, and continuous SATED sensitivity analyses used linear regression with robust standard errors.

Model convergence and separation were checked for logistic models. Calibration was assessed descriptively using Hosmer–Lemeshow goodness-of-fit tests where model assumptions allowed. Collinearity was assessed using variance inflation factors and pairwise correlations among HAE-AS, AECT, and AE-QoL domains. ORs, RRRs, beta coefficients, 95% CIs, exact two-sided P values, and model-specific Ns are reported. Because multiple sleep outcomes and predictors were evaluated, P values were interpreted descriptively rather than as confirmatory multiplicity-adjusted tests. Statistical significance was defined as P < 0.05.

### Ethical statement

The study adhered to the Declaration of Helsinki. The protocol was reviewed and approved by the Committee of Ethics in Research on Human Beings (CEISH) in Guayaquil, Ecuador (Approval Code: HCK-CEISH-21–003). Informed consent was obtained electronically from all participants; the survey could not proceed without an affirmative selection of the consent option Table 1.

**Table 1:** Participant flow diagram

Survey landing page views	1,070
Responses initiated	635
Completed forms	300 (150 physician forms and 150 patient forms)
Paired patient records after linkage	150
Excluded records with missing or implausible critical analytic variables	11
Final analytic sample	139 participants

## Results

### Patient demographics

In total, 139 patients with HAE were included in the final analytic sample (Table 2). The mean age was 42.1 (14.7) years, the mean BMI was 26.6 (4.3) kg/m2, and the mean neck circumference was 35.0 (7.42) cm. Most participants were female (95/139, 68.4%) and had HAE-C1INH type 1 (89/139, 64.0%). Stress and trauma were reported as triggers by 131/139 (94.2%) and 92/139 (66.2%) participants, respectively; menstruation was reported by 61/139 (43.9%) participants. Percentages were calculated using the final analytic denominator unless otherwise stated Tables 3 and 4.

**Table 2:** Demographic and disease characteristics

Variable	Overall sample (N = 139)
Demographics	
Age, mean (SD)	42.1 (14.7)
BMI, mean (SD)	26.6 (4.3)
Neck circumference, cm, mean (SD)	35.0 (7.42)
Female, n (%)	95 (68.4%)
Employment Type, n (%)	
Full-time	57 (41.0%)
Part-time	17 (12.2%)
Self-employed worker	32 (23%)
Other	21 (15.1%)
Unemployed	10 (7.2%)
Student	12 (8.6%)
Hae Disease/Assessments	
HAE Subtype, n (%)	
HAE-C1INH type 1	89 (64.0%)
HAE-C1INH type 2	17 (12.2%)
HAE-nC1INH, FXII mutation	4 (2.9%)
HAE-nC1INH, other/unspecified known mutation	4 (2.9%)
HAE-nC1INH, unknown mutation/cause	8 (5.8%)
Subtype not available	17 (12.2%)
Time of day when HAE causes most discomfort, n (%)	
Morning	16 (11.5%)
Afternoon	5 (3.6%)
Nights	26 (18.7%)
Only while sleeping	2 (1.4%)
Any moment of the day	105 (75.5%)
HAE management, n (%)	
None	27 (19.4%)
Only On-demand	56 (40.3%)
Only Long-term Prophylaxis	9 (6.5%)
Both (on-demand and long-term prophylaxis)	47 (33.8%)
Long-term prophylaxis management, n (%)	56 (40.3%)
IV Human Plasma-Derived C1 Esterase Inhibitor (ie, Cinryze®)	7 (5.0%)
SC Human Plasma-Derived C1 Esterase Inhibitor (ie, HAEGARDA®)	6 (4.3%)
Human IgG1 monoclonal antibody to kallikrein (ie, lanadelumab)	18 (12.9%)
Androgen Derivatives (i.e. danazol)	4 (2.9%)
Antifibrinolytics agents (i.e. tranexamic acid)	10 (7.2%)
Kallikrein inhibitor (i.e. berotralstat)	0
Second-generation antihistamines	3 (2.2%)
On-demand management, n (%)	103 (74.1%)
Plasma-derived C1- esterase inhibitor (ie,. berinert, **c**inryze)	26 (18.7%)
Inhibitor of plasma kallikrein (ie, ecallantide)	0
Recombinant C1-INH (rh-C1-INH) (ie, ruconest)	0
Inhibitor of the β2 bradykinin receptor (ie, icatibant)	58 (41.7%)
AE-QoL mean (SD)	
Overall	51.788 (19.79)
Fears/shame domain	58.723 (25.15)
Fatigue/Mood domain	51.511 (23.99)
Functioning domain	46.897 (23.99)
Nutrition domain	41.457 (28.99)
AECT	
Overall, mean (SD)	9.309 (2.71)
Uncontrolled, n (%)	71 (51.1%)
Controlled, n (%)	68 (48.9%)
HAE-AS	
Overall, mean (SD)	11.820 (4.7)
Severe activity, n (%)	78 (56.1%)
Non severe activity, n (%)	61 (43.9%)
Sleep dısorders/assessments	
Sleep disorder, n (%)	
Insomnia	75 (54.0%)
GSAQ-defined sleep apnea symptoms	39 (28.1%)
Hypersomnolence	31 (22.3%)
SWSD	24 (17.3%)
RLS/PLMD	21 (15.1%)
REM-SBD	7 (5.0%)
Parasomnia	7 (5.0%)
OSA risk assessed by STOP-Bang, n (%)	
Low risk	91 (65.5%)
Moderate-to-high risk	48 (34.5%)
Sleep quality assessed by SATED	
Overall, mean (SD**)**	5.46 (1.87)
Poor, n (%)	77 (55.4%)
Intermediate, n (%)	53 (38.1%)
Good sleep health, n (%)	9 (6.5%)
**Comorbidities, n (%)**	
Rhinitis	21 (15.1%)
Gastritis	15 (10.8%)
Hypertension	12 (8.6%)
Drug allergy	10 (7.2%)
Polycystic ovary syndrome	7 (5.0%)
Type 2 diabetes	4 (2.9%)
None	17 (12.2%)

Values are n (%) unless otherwise indicated. Percentages are based on the overall analytic sample (N = 139) unless otherwise stated. Employment status, HAE triggers, treatment products, and comorbidities were non-mutually exclusive survey items and are therefore not expected to sum to 100%. No imputation was performed. AECT = Angioedema Control Test; AE-QoL = Angioedema Quality of Life; BMI = body mass index; CI = confidence interval; HAE = hereditary angioedema; HAE-AS = Hereditary Angioedema Activity Score; HAE-C1INH = HAE due to C1 inhibitor deficiency; HAE-nC1INH = HAE with normal C1 inhibitor; IV = intravenous; OSA = obstructive sleep apnea; PLMD = periodic limb movement disorder; REM-SBD = rapid eye movement sleep behavior disorder; RLS = restless legs syndrome; SATED = Satisfaction, Alertness, Timing, Efficiency, and Duration; SC = subcutaneous; STOP-Bang = Snoring, Tiredness, Observed apnea, high blood pressure, body mass index, age, neck circumference, and gender; SWSD = shift work sleep disorder

**Table 3:** Age-, sex-, and BMI-adjusted demographic predictors of sleep outcomes

Outcome	Predictor	OR (95% CI)	P value
Insomnia	Age	1.00 (0.97–1.02)	0.687
Insomnia	Male sex	0.40 (0.19–0.83)	0.014
Insomnia	BMI	1.02 (0.94–1.10)	0.677
GSAQ-defined sleep apnea symptoms	Age	1.02 (0.99–1.05)	0.127
GSAQ-defined sleep apnea symptoms	Male sex	1.16 (0.51–2.62)	0.723
GSAQ-defined sleep apnea symptoms	BMI	1.12 (1.02–1.23)	0.014
Hypersomnolence	Age	1.00 (0.98–1.03)	0.844
Hypersomnolence	Male sex	0.56 (0.22–1.42)	0.223
Hypersomnolence	BMI	1.02 (0.93–1.12)	0.691
SWSD	Age	0.98 (0.95–1.01)	0.247
SWSD	Male sex	0.84 (0.32–2.23)	0.733
SWSD	BMI	0.98 (0.88–1.09)	0.722
RLS/PLMD	Age	0.98 (0.94–1.01)	0.174
RLS/PLMD	Male sex	1.05 (0.38–2.85)	0.927
RLS/PLMD	BMI	1.07 (0.96–1.19)	0.223
REM-SBD	Age	0.96 (0.90–1.02)	0.171
REM-SBD	Male sex	1.62 (0.33–7.95)	0.550
REM-SBD	BMI	0.91 (0.75–1.12)	0.382
Parasomnia	Age	0.96 (0.90–1.02)	0.171
Parasomnia	Male sex	1.62 (0.33–7.95)	0.550
Parasomnia	BMI	0.91 (0.75–1.12)	0.382
Poor sleep health (SATED < = 5)	Age	0.99 (0.97–1.02)	0.533
Poor sleep health (SATED < = 5)	Male sex	0.71 (0.34–1.46)	0.350
Poor sleep health (SATED < = 5)	BMI	1.07 (0.99–1.16)	0.104
STOP-Bang moderate-to-high OSA risk	Age	1.06 (1.03–1.09)	0.00012
STOP-Bang moderate-to-high OSA risk	Male sex	0.59 (0.25–1.39)	0.226
STOP-Bang moderate-to-high OSA risk	BMI	1.13 (1.03–1.25)	0.0097

All models used N = 139 complete cases. ORs and 95% CIs are from logistic regression models with age, sex, and BMI included simultaneously. Age is modeled per year, and BMI is modeled per kg/m2. Exact two-sided P values are reported and interpreted descriptively because analyses were exploratory and multiple outcomes were evaluated. CI = confidence interval; GSAQ = Global Sleep Assessment Questionnaire; OR = odds ratio; REM-SBD = rapid eye movement sleep behavior disorder; RLS/PLMD = restless legs syndrome/periodic limb movement disorder; SATED = Satisfaction, Alertness, Timing, Efficiency, and Duration; STOP-Bang = Snoring, Tiredness, Observed apnea, high blood pressure, body mass index, age, neck circumference, and gender; SWSD = shift work sleep disorder

**Table 4:** Clinical activity and AE-QoL predictors of sleep outcomes in age-, sex-, and BMI-adjusted models

Outcome	Predictor	OR (95% CI)	P value
Insomnia	Severe HAE activity	2.62 (1.28–5.35)	0.0083
Insomnia	AE-QoL functioning	1.01 (0.99–1.02)	0.236
Insomnia	AE-QoL fatigue/mood	1.09 (1.06–1.12)	1.78e-8
Insomnia	AE-QoL fears/shame	1.03 (1.02–1.05)	0.000084
Insomnia	AE-QoL nutrition	1.00 (0.99–1.01)	0.699
GSAQ-defined sleep apnea symptoms	Severe HAE activity	2.40 (1.03–5.59)	0.042
GSAQ-defined sleep apnea symptoms	AE-QoL functioning	1.03 (1.01–1.05)	0.0028
GSAQ-defined sleep apnea symptoms	AE-QoL fatigue/mood	1.02 (1.00–1.04)	0.022
GSAQ-defined sleep apnea symptoms	AE-QoL fears/shame	1.02 (1.00–1.04)	0.035
GSAQ-defined sleep apnea symptoms	AE-QoL nutrition	1.03 (1.01–1.05)	0.00039
Hypersomnolence	Severe HAE activity	0.70 (0.31–1.61)	0.405
Hypersomnolence	AE-QoL functioning	1.02 (1.00–1.04)	0.081
Hypersomnolence	AE-QoL fatigue/mood	1.07 (1.04–1.10)	5.81e-7
Hypersomnolence	AE-QoL fears/shame	1.01 (1.00–1.03)	0.096
Hypersomnolence	AE-QoL nutrition	1.01 (0.99–1.02)	0.286
SWSD	Severe HAE activity	1.05 (0.42–2.63)	0.912
SWSD	AE-QoL functioning	1.01 (0.99–1.03)	0.273
SWSD	AE-QoL fatigue/mood	1.04 (1.01–1.06)	0.0015
SWSD	AE-QoL fears/shame	1.01 (0.99–1.03)	0.168
SWSD	AE-QoL nutrition	1.01 (1.00–1.03)	0.163
RLS/PLMD	Severe HAE activity	5.85 (1.59–21.62)	0.0080
RLS/PLMD	AE-QoL functioning	1.03 (1.00–1.05)	0.022
RLS/PLMD	AE-QoL fatigue/mood	1.05 (1.03–1.08)	0.000077
RLS/PLMD	AE-QoL fears/shame	1.04 (1.01–1.06)	0.0026
RLS/PLMD	AE-QoL nutrition	1.02 (1.00–1.04)	0.055
REM-SBD	Severe HAE activity	2.19 (0.39–12.32)	0.375
REM-SBD	AE-QoL functioning	1.02 (0.98–1.06)	0.315
REM-SBD	AE-QoL fatigue/mood	1.02 (0.98–1.05)	0.364
REM-SBD	AE-QoL fears/shame	1.01 (0.98–1.05)	0.491
REM-SBD	AE-QoL nutrition	1.01 (0.99–1.04)	0.363
Parasomnia	Severe HAE activity	2.19 (0.39–12.32)	0.375
Parasomnia	AE-QoL functioning	1.02 (0.98–1.06)	0.315
Parasomnia	AE-QoL fatigue/mood	1.02 (0.98–1.05)	0.364
Parasomnia	AE-QoL fears/shame	1.01 (0.98–1.05)	0.491
Parasomnia	AE-QoL nutrition	1.01 (0.99–1.04)	0.363
Poor sleep health (SATED < = 5)	Severe HAE activity	1.62 (0.80–3.26)	0.179
Poor sleep health (SATED < = 5)	AE-QoL functioning	1.01 (0.99–1.02)	0.428
Poor sleep health (SATED < = 5)	AE-QoL fatigue/mood	1.03 (1.01–1.04)	0.0013
Poor sleep health (SATED < = 5)	AE-QoL fears/shame	1.02 (1.01–1.04)	0.0052
Poor sleep health (SATED < = 5)	AE-QoL nutrition	1.00 (0.99–1.01)	0.978
STOP-Bang moderate-to-high OSA risk	Severe HAE activity	2.37 (1.01–5.55)	0.047
STOP-Bang moderate-to-high OSA risk	AE-QoL functioning	1.01 (1.00–1.03)	0.109
STOP-Bang moderate-to-high OSA risk	AE-QoL fatigue/mood	1.04 (1.02–1.06)	0.000093
STOP-Bang moderate-to-high OSA risk	AE-QoL fears/shame	1.01 (1.00–1.03)	0.090
STOP-Bang moderate-to-high OSA risk	AE-QoL nutrition	1.02 (1.00–1.03)	0.033

Each row corresponds to a separate logistic regression model adjusted for age, sex, and BMI; all models used N = 139 complete cases. ORs for AE-QoL domains are per 1-point increase in domain score. Exact two-sided P values are reported and interpreted descriptively. AECT full-model estimates are reported in Table 5. AE-QoL = Angioedema Quality of Life; BMI = body mass index; CI = confidence interval; GSAQ = Global Sleep Assessment Questionnaire; HAE = hereditary angioedema; OR = odds ratio; REM-SBD = rapid eye movement sleep behavior disorder; RLS/PLMD = restless legs syndrome/periodic limb movement disorder; SATED = Satisfaction, Alertness, Timing, Efficiency, and Duration; STOP-Bang = Snoring, Tiredness, Observed apnea, high blood pressure, body mass index, age, neck circumference, and gender; SWSD = shift work sleep disorder

**Table 5:** Exploratory full models, sensitivity analyses, and COMISA correlates

Analysis	Outcome	Predictor	Estimate (95% CI), P
Exploratory full model	Insomnia	Uncontrolled AECT status	OR = 0.54 (0.18–1.65), P = 0.277
Exploratory full model	GSAQ-defined sleep apnea symptoms	Uncontrolled AECT status	OR = 0.51 (0.19–1.37), P = 0.182
Exploratory full model	Hypersomnolence	Uncontrolled AECT status	OR = 1.07 (0.31–3.69), P = 0.915
Exploratory full model	SWSD	Uncontrolled AECT status	OR = 0.59 (0.19–1.83), P = 0.361
Exploratory full model	RLS/PLMD	Uncontrolled AECT status	OR = 1.68 (0.48–5.90), P = 0.420
Exploratory full model	STOP-Bang moderate-to-high OSA risk	Uncontrolled AECT status	OR = 0.66 (0.25–1.76), P = 0.407
STOP-Bang sensitivity	Low/intermediate/high risk strata	Severe HAE activity	OR = 2.37 (1.05–5.34), P = 0.037
STOP-Bang sensitivity	Low/intermediate/high risk strata	AE-QoL fatigue/mood	OR = 1.04 (1.02–1.06), P = 0.000087
SATED sensitivity	Continuous SATED score	Severe HAE activity	beta = −0.360 (−0.989 to 0.269), P = 0.262
SATED sensitivity	Continuous SATED score	AE-QoL fatigue/mood	beta = −0.030 (−0.043 to −0.018), P = 1.34e-6
SATED sensitivity	Continuous SATED score	AE-QoL fears/shame	beta = −0.019 (−0.031 to −0.007), P = 0.0018
COMISA correlates	COMISA	Severe HAE activity	OR = 2.86 (1.12–7.29), P = 0.028
COMISA correlates	COMISA	AE-QoL fatigue/mood	OR = 1.07 (1.04–1.10), P = 1.19e-6
COMISA correlates	COMISA	AE-QoL fears/shame	OR = 1.03 (1.01–1.05), P = 0.006

All models used N = 139 complete cases. Exploratory full models included severe HAE activity, AECT control status, AE-QoL domains, age, sex, and BMI. STOP-Bang sensitivity analyses used ordinal logistic regression with the standard low, intermediate, and high risk strata. SATED sensitivity analyses used linear regression with robust standard errors. COMISA was defined as concurrent GSAQ-defined insomnia and STOP-Bang moderate-to-high OSA risk. AECT = Angioedema Control Test; AE-QoL = Angioedema Quality of Life; BMI = body mass index; CI = confidence interval; COMISA = comorbid insomnia and sleep apnea; GSAQ = Global Sleep Assessment Questionnaire; HAE = hereditary angioedema; OR = odds ratio; RLS/PLMD = restless legs syndrome/periodic limb movement disorder; SATED = Satisfaction, Alertness, Timing, Efficiency, and Duration; STOP-Bang = Snoring, Tiredness, Observed apnea, high blood pressure, body mass index, age, neck circumference, and gender; SWSD = shift work sleep disorder

The mean overall HAE-AS score was 11.8 (4.7), and 78 participants (56.1%) had severe HAE activity. The mean AECT score was 9.3 (2.7), with uncontrolled disease in 71 participants (51.1%). The mean AE-QoL total score was 51.8 (19.8); the fears/shame domain was the most affected domain, followed by fatigue/mood, functioning, and nutrition.

The overall mean SATED score was 5.46 (1.87), and only 9 participants (6.5%) had good sleep health. Insomnia was the most prevalent sleep disorder (75/139, 54.0%), followed by STOP-Bang moderate-to-high OSA risk (48/139, 34.5%), GSAQ-defined sleep apnea symptoms (39/139, 28.1%), hypersomnolence (31/139, 22.3%), SWSD (24/139, 17.3%), and RLS/PLMD (21/139, 15.1%).

In supplemental analyses, insomnia was more frequent in participants with severe HAE activity than in those with non-severe activity (65.4% vs. 39.3%, P = 0.002), as was RLS/PLMD (23.1% vs. 4.9%, P = 0.003). GSAQ-defined sleep apnea symptoms were also more frequent in participants with severe activity (34.6% vs. 19.7%, P = 0.050). STOP-Bang moderate-to-high OSA risk was 41.0% in participants with severe activity and 26.2% in those with non-severe activity (P = 0.070).

### Regression analysis

All adjusted regression models used the final analytic sample of 139 participants. In age-, sex-, and BMI-adjusted models, male sex was associated with lower odds of insomnia compared with female sex (OR = 0.40, 95% CI: 0.19–0.83, P = 0.014). BMI was associated with higher odds of GSAQ-defined sleep apnea symptoms (OR = 1.12 per kg/m2, 95% CI: 1.02–1.23, P = 0.014). Age and BMI were associated with higher odds of STOP-Bang moderate-to-high OSA risk (age: OR = 1.06 per year, 95% CI: 1.03–1.09, P = 0.00012; BMI: OR = 1.13 per kg/m2, 95% CI: 1.03–1.25, P = 0.0097).

In clinical models adjusted for age, sex, and BMI, severe HAE activity was associated with higher odds of insomnia (OR = 2.62, 95% CI: 1.28–5.35, P = 0.0083), RLS/PLMD (OR = 5.85, 95% CI: 1.59–21.62, P = 0.0080), GSAQ-defined sleep apnea symptoms (OR = 2.40, 95% CI: 1.03–5.59, P = 0.042), and STOP-Bang moderate-to-high OSA risk (OR = 2.37, 95% CI: 1.01–5.55, P = 0.047).

Higher AE-QoL fatigue/mood scores were associated with insomnia (OR = 1.09 per 1-point increase, 95% CI: 1.06–1.12, P = 1.78e-8), hypersomnolence (OR = 1.07, 95% CI: 1.04–1.10, P = 5.81e-7), SWSD (OR = 1.04, 95% CI: 1.01–1.06, P = 0.0015), RLS/PLMD (OR = 1.05, 95% CI: 1.03–1.08, P = 0.000077), and STOP-Bang moderate-to-high OSA risk (OR = 1.04, 95% CI: 1.02–1.06, P = 0.000093). Higher AE-QoL fears/shame scores were associated with insomnia (OR = 1.03, 95% CI: 1.02–1.05, P = 0.000084), GSAQ-defined sleep apnea symptoms (OR = 1.02, 95% CI: 1.00–1.04, P = 0.035), and RLS/PLMD (OR = 1.04, 95% CI: 1.01–1.06, P = 0.0026).

In exploratory full models including HAE activity, AECT control status, AE-QoL domains, age, sex, and BMI, AECT control status did not show a consistent independent association with the main sleep outcomes. Uncontrolled AECT status was not independently associated with insomnia (OR = 0.54, 95% CI: 0.18–1.65, P = 0.277), GSAQ-defined sleep apnea symptoms (OR = 0.51, 95% CI: 0.19–1.37, P = 0.182), hypersomnolence (OR = 1.07, 95% CI: 0.31–3.69, P = 0.915), SWSD (OR = 0.59, 95% CI: 0.19–1.83, P = 0.361), RLS/PLMD (OR = 1.68, 95% CI: 0.48–5.90, P = 0.420), or STOP-Bang moderate-to-high OSA risk (OR = 0.66, 95% CI: 0.25–1.76, P = 0.407). All primary logistic models converged. Variance inflation factors in the exploratory full models were below 3, although HAE-AS, AECT, and AE-QoL domains remain conceptually overlapping measures of disease burden.

Sensitivity analyses supported the main findings. When STOP-Bang was analyzed using the standard low, intermediate, and high risk strata, 91 participants (65.5%) were low risk, 38 (27.3%) were intermediate risk, and 10 (7.2%) were high risk. In ordinal logistic models adjusted for age, sex, and BMI, severe HAE activity was associated with higher STOP-Bang risk strata (OR = 2.37, 95% CI: 1.05–5.34, P = 0.037), as was higher AE-QoL fatigue/mood score (OR = 1.04 per 1-point increase, 95% CI: 1.02–1.06, P = 0.000087). When SATED was analyzed as a continuous 0–10 score, higher AE-QoL fatigue/mood score was associated with lower sleep health score (beta = −0.030, 95% CI: −0.043 to −0.018, P = 1.34e-6), and higher AE-QoL fears/shame score was also associated with lower sleep health score (beta = −0.019, 95% CI: −0.031 to −0.007, P = 0.0018). Severe HAE activity was not significantly associated with continuous SATED score after adjustment (beta = −0.360, 95% CI: −0.989 to 0.269, P = 0.262).

COMISA was present in 34 of 139 participants (24.5%). In age-, sex-, and BMI-adjusted models, severe HAE activity was associated with higher odds of COMISA (OR = 2.86, 95% CI: 1.12–7.29, P = 0.028). Higher AE-QoL fatigue/mood scores (OR = 1.07 per 1-point increase, 95% CI: 1.04–1.10, P = 1.19e-6) and fears/shame scores (OR = 1.03, 95% CI: 1.01–1.05, P = 0.006) were also associated with COMISA.

## Discussion

This multicenter survey provides evidence that sleep disorders are common among Latin American patients with HAE and that the burden of sleep symptoms is associated with HAE activity and quality-of-life impairment. More than half of participants reported insomnia, approximately one-third had STOP-Bang moderate-to-high OSA risk, and one-quarter met the screening definition for COMISA. These findings should be interpreted as associations because the cross-sectional design does not establish directionality or causality.

The literature addressing sleep health in HAE remains limited. Prior registry and single-center data have suggested that sleep disturbance occurs in patients with HAE-C1INH, but the specific sleep phenotypes and their clinical correlates have not been well described. Our findings extend this work by using structured sleep instruments and by linking sleep outcomes to HAE activity and AE-QoL domains. Consistent with evidence that common lifestyle stimulants influence self-reported sleep quality in adult populations [65], caffeine intake should also be considered as a modifiable factor when interpreting sleep complaints in this cohort.

The difference between GSAQ-defined sleep apnea symptoms (28.1%) and STOP-Bang moderate-to-high OSA risk (34.5%) reflects the distinct constructs captured by these instruments. The GSAQ focuses on self-reported symptoms, whereas STOP-Bang estimates screening-based OSA risk using clinical and anthropometric predictors such as age, BMI, neck circumference, and sex. Therefore, these two estimates should not be interpreted as conflicting measures of the same outcome.

COMISA was defined as the co-occurrence of GSAQ-defined insomnia and STOP-Bang moderate-to-high OSA risk in the same participant. This screening-based definition does not indicate polysomnography-confirmed OSA, but it identifies a subgroup with overlapping insomnia symptoms and elevated OSA risk [66–70]. The cardiometabolic implications of COMISA are clinically relevant in the broader sleep literature, but our data do not permit inference about cardiovascular events or thrombotic outcomes in HAE. These findings should therefore be viewed as hypothesis-generating and as support for future longitudinal studies rather than as evidence for preventive cardiovascular interventions. The overlap between fatigue and functional status observed in other chronic respiratory conditions [71] further supports a broader assessment of symptom burden in patients with overlapping insomnia and OSA risk.

Disease control measured by AECT did not show a consistent independent association with sleep outcomes after simultaneous adjustment for HAE activity, AE-QoL domains, age, sex, and BMI. This finding should be interpreted cautiously because HAE-AS, AECT, and AE-QoL domains capture overlapping aspects of disease burden. Although variance inflation factors were below conventional thresholds, conceptual overlap among these measures limits strong claims about independent effects.

These findings support the consideration of sleep screening in the care of patients with HAE, particularly those who experience high disease activity or significant fatigue, mood issues, and impairments related to fears or shame. The GSAQ, STOP-Bang, and SATED instruments provide complementary information and may help identify patients who need further sleep evaluation. However, clinical implementation should be guided by prospective validation and by standard diagnostic pathways for sleep disorders. Emerging digital decision-support tools [72] may further facilitate PROM-based screening pathways in clinical practice. Clinically, incorporating brief validated sleep screening into routine HAE follow-up visits could help identify patients who would benefit from formal sleep evaluation and earlier intervention, particularly those with high disease activity or marked quality-of-life impairment.

### Strengths

This study has several strengths. It is, to our knowledge, the first multicenter investigation to characterize sleep disorders across Latin American patients with HAE, drawing on a paired physician–patient data collection model in which objective clinical variables were confirmed by the treating physician while subjective measures were reported directly by the patient, reducing recall bias and improving diagnostic precision. The use of multiple validated, complementary sleep instruments (GSAQ, STOP-Bang, SATED) alongside disease-specific measures (HAE-AS, AECT, AE-QoL) allowed sleep outcomes to be examined in relation to both general demographic and HAE-specific predictors within consistently adjusted models across five countries.

### Study limitations

Several limitations should be acknowledged. First, the cross-sectional design precludes inference about directionality or causal relationships between HAE activity and sleep outcomes. Second, reliance on an online survey may introduce recall bias and may have contributed to under-reporting of comorbidities. Although the paired physician–patient response model improved diagnostic accuracy, we did not have direct access to primary hospital records or independent laboratory confirmation for all participants. Third, validated Spanish versions were not available across all participating countries for the HAE-AS, GSAQ, and SATED; ad hoc translations may affect psychometric precision. Fourth, the three-level SATED classification was used for descriptive clinical interpretability and is not a formally standardized categorization, although continuous SATED sensitivity analyses were also performed. Fifth, potential center- and country-level heterogeneity may remain because center identifiers were not retained in the anonymized analytic dataset available for modeling. Sixth, although the survey instrument queried psychiatric and psychological comorbidities such as anxiety and depression, participants who reported these conditions were disproportionately less likely to complete the core sleep-disorder questionnaires and were therefore excluded from the analytic sample under the missing-data criterion; this attrition may have led to underrepresentation of patients with psychiatric comorbidity and could bias prevalence estimates of sleep disorders in this population. Seventh, because the sample was drawn from Latin American HAE reference centers, findings may not generalize directly to HAE populations with different demographic, healthcare-access, or cultural characteristics. Finally, the number of sleep outcomes and clinical predictors increases the risk of false-positive findings, and some outcomes had sparse event counts. P values should therefore be interpreted descriptively.

Future research should prioritize prospective longitudinal designs, formal cultural validation of HAE activity and sleep instruments across Latin American settings, and age- and BMI-matched comparisons to clarify whether HAE activity contributes independently to sleep-disorder burden. Behavioral and exercise-based interventions, including respiratory training, structured walking programs, and lifestyle modification, have improved apnea–hypopnea index, daytime sleepiness, and self-reported sleep quality in other OSA-associated populations [73–76], suggesting that similar intervention strategies may warrant future evaluation in patients with HAE.

In conclusion, this exploratory multicenter study found that sleep disorders were common in patients with HAE and were associated with severe HAE activity and impaired quality of life. Targeted sleep screening may be a useful addition to HAE care pathways if these findings are confirmed by prospective studies.

## Supplementary Information

Below is the link to the electronic supplementary material.

Supplementary file1 (DOCX 27 KB)

## Data Availability

The data supporting the findings of this study are available from the corresponding author upon reasonable request.

## Abbreviations

HAE: Hereditary Angioedema
C1INH: C1 inhibitor
GSAQ: Global Sleep Assessment Questionnaire
SATED: Satisfaction, Alertness, Timing, Efficiency, and Duration
STOP-Bang: Snoring, Tiredness, Observed apnea, high blood pressure, body mass index, age, neck circumference, and gender
OSA: Obstructive Sleep Apnea
HAE-AS: Hereditary Angioedema Activity Score
AECT: Angioedema Control Test
AE-QoL: Angioedema Quality of Life
HAE-C1INH-Type 1: Hereditary Angioedema Type 1 with insufficient levels of C1 inhibitor.
HAE-C1INH-Type 2: Hereditary Angioedema Type 1 with dysfunctional levels of C1 inhibitor.
HAE-nC1INH: Hereditary Angioedema with normal levels of C1 inhibitor.
QoL: Quality of Life
REM: Rapid Eye Movement
RLS: Restless Leg Syndrome
PLMD: Periodic Limb Movement Disorder
SWSD: Shift Work Sleep Disorder
REM-SD: Rapid Eye Movement Sleep Disorders
REM-SBD: REM sleep behavior disorder
COMISA: COMorbid Insomnia and Sleep Apnea
MACE: Major adverse cardiovascular events
CVD: Cardiovascular disease
VTE: Venous thromboembolism
PROMs: Patient-reported outcome measures
UCARE: Urticaria Centres of Reference and Excellence
GA^2^LEN: Global Allergy and Asthma European Network

## References

[1] Christiansen SC, Wilmot J, Castaldo AJ, Zuraw BL (2023) The US Hereditary Angioedema Association Scientific Registry: hereditary angioedema demographics, disease severity, and comorbidities. Ann Allergy Asthma Immunol 131(6):766-774.e768 10.1016/j.anai.2023.08.012 37619776

[2] Csuka D, Veszeli N, Imreh É, Zotter Z, Skopál J, Prohászka Z et al (2015) Comprehensive study into the activation of the plasma enzyme systems during attacks of hereditary angioedema due to C1-inhibitor deficiency. Orphanet J Rare Dis 10:132 10.1186/s13023-015-0351-5 PMC4600308 26452350

[3] Grover SP, Sundler Björkman L, Egesten A, Moll S, Mackman N (2022) Hereditary angioedema is associated with an increased risk of venous thromboembolism. J Thromb Haemost 20(11):2703–2706 10.1111/jth.15870 PMC12541594 36053174

[4] Grover SP, Kawano T, Wan J, Tanratana P, Polai Z, Shim YJ et al (2023) C1 inhibitor deficiency enhances contact pathway-mediated activation of coagulation and venous thrombosis. Blood 141(19):2390–2401 10.1182/blood.2022018849 PMC10273165 36701760

[5] Betschel S, Badiou J, Binkley K, Borici-Mazi R, Hébert J, Kanani A et al (2019) The International/Canadian Hereditary Angioedema Guideline. Allergy Asthma Clin Immunol 15:72 10.1186/s13223-019-0376-8 PMC6878678 31788005

[6] Sinnathamby ES, Issa PP, Roberts L, Norwood H, Malone K, Vemulapalli H et al (2023) Hereditary angioedema: diagnosis, clinical implications, and pathophysiology. Adv Ther 40(3):814–827 10.1007/s12325-022-02401-0 PMC9988798 36609679

[7] Reshef A, Buttgereit T, Betschel SD, Caballero T, Farkas H, Grumach AS et al (2024) Definition, acronyms, nomenclature, and classification of angioedema (DANCE): AAAAI, ACAAI, ACARE, and APAAACI DANCE consensus. J Allergy Clin Immunol 154(2):398-411.e391 10.1016/j.jaci.2024.03.024 38670233

[8] Busse PJ, Christiansen SC (2020) Hereditary angioedema. N Engl J Med 382(12):1136–1148 10.1056/NEJMra1808012 32187470

[9] Wilkerson RG, Moellman JJ (2022) Hereditary angioedema. Emerg Med Clin North Am 40(1):99–118 10.1016/j.emc.2021.09.002 34782094

[10] Hews-Girard J, Goodyear MD (2021) Psychosocial burden of type 1 and 2 hereditary angioedema: a single-center Canadian cohort study. Allergy Asthma Clin Immunol 17(1):61 10.1186/s13223-021-00563-0 PMC8244202 34187550

[11] Mendivil J, Murphy R, de la Cruz M, Janssen E, Boysen HB, Jain G et al (2021) Clinical characteristics and burden of illness in patients with hereditary angioedema: findings from a multinational patient survey. Orphanet J Rare Dis 16(1):94 10.1186/s13023-021-01717-4 PMC7893968 33602292

[12] Bygum A, Aygören-Pürsün E, Beusterien K, Hautamaki E, Sisic Z, Wait S et al (2015) Burden of illness in hereditary angioedema: a conceptual model. Acta Derm Venereol 95(6):706–710 10.2340/00015555-2014 25394853

[13] Lumry WR, Castaldo AJ, Vernon MK, Blaustein MB, Wilson DA, Horn PT (2010) The humanistic burden of hereditary angioedema: Impact on health-related quality of life, productivity, and depression. Allergy Asthma Proc 31(5):407–414 10.2500/aap.2010.31.3394 20929608

[14] Aygören-Pürsün E, Bygum A, Beusterien K, Hautamaki E, Sisic Z, Wait S et al (2014) Socioeconomic burden of hereditary angioedema: results from the hereditary angioedema burden of illness study in Europe. Orphanet J Rare Dis 9:99 10.1186/1750-1172-9-99 PMC4105891 24996814

[15] Klingman KJ, Jungquist CR, Perlis ML (2017) Questionnaires that screen for multiple sleep disorders. Sleep Med Rev 32:37–44 10.1016/j.smrv.2016.02.004 PMC6543842 27013458

[16] Bin YS, Marshall NS, Glozier N (2012) Secular trends in adult sleep duration: a systematic review. Sleep Med Rev 16(3):223–230 10.1016/j.smrv.2011.07.003 22075214

[17] Medicine. Io (2006) Sleep disorders and sleep deprivation: an unmet public health problem. The National Academies Press, Washington, DC, USA 20669438

[18] Hafner M, Stepanek M, Taylor J, Troxel WM, van Stolk C (2017) Why Sleep Matters-The Economic Costs of Insufficient Sleep: A Cross-Country Comparative Analysis. Rand Health Q 6(4):11 10.7249/rhq-06-04-11 PMC5627640 28983434

[19] Luyster FS, Baniak LM, Imes CC, Jeon B, Morris JL, Orbell S et al (2024) Association of comorbid obstructive sleep apnea and insomnia with risk of major adverse cardiovascular events in sleep medicine center patients. Sleep Health 10(3):335–341 10.1016/j.sleh.2024.03.001 38704352

[20] Spiegel K, Leproult R, Van Cauter E (1999) Impact of sleep debt on metabolic and endocrine function. Lancet 354(9188):1435–1439 10.1016/S0140-6736(99)01376-8 10543671

[21] Durmer JS, Dinges DF (2005) Neurocognitive consequences of sleep deprivation. Semin Neurol 25(1):117–129 10.1055/s-2005-867080 15798944

[22] Irwin MR (2015) Why sleep is important for health: a psychoneuroimmunology perspective. Annu Rev Psychol 66:143–172 10.1146/annurev-psych-010213-115205 PMC4961463 25061767

[23] Coskun BI (2018) Sleep impairment: an obstacle to achieve optimal quality of life in rheumatoid arthritis. Rheumatol Int 38(12):2183–2192 10.1007/s00296-018-4155-5 30206671

[24] Gottlieb DJ, Yenokyan G, Newman AB, O’Connor GT, Punjabi NM, Quan SF et al (2010) Prospective study of obstructive sleep apnea and incident coronary heart disease and heart failure: the sleep heart health study. Circulation 122(4):352–360 10.1161/CIRCULATIONAHA.109.901801 PMC3117288 20625114

[25] Bhuniya S, Goyal M, Chowdhury N, Mishra P (2022) Intermittent hypoxia and sleep disruption in obstructive sleep apnea increase serum tau and amyloid-beta levels. J Sleep Res 31(5):e13566 10.1111/jsr.13566 35165967

[26] Canivet C, Nilsson PM, Lindeberg SI, Karasek R, Östergren PO (2014) Insomnia increases risk for cardiovascular events in women and in men with low socioeconomic status: a longitudinal, register-based study. J Psychosom Res 76(4):292–299 10.1016/j.jpsychores.2014.02.001 24630179

[27] Breslau N, Roth T, Rosenthal L, Andreski P (1996) Sleep disturbance and psychiatric disorders: a longitudinal epidemiological study of young adults. Biol Psychiatry 39(6):411–418 10.1016/0006-3223(95)00188-3 8679786

[28] Palma JA, Urrestarazu E, Iriarte J (2013) Sleep loss as risk factor for neurologic disorders: a review. Sleep Med 14(3):229–236 10.1016/j.sleep.2012.11.019 23352029

[29] Banks S, Dinges DF (2007) Behavioral and physiological consequences of sleep restriction. J Clin Sleep Med 3(5):519–528 PMC1978335 17803017

[30] Reutrakul S, Van Cauter E (2018) Sleep influences on obesity, insulin resistance, and risk of type 2 diabetes. Metabolism 84:56–66 10.1016/j.metabol.2018.02.010 29510179

[31] Feng X, Liu Q, Li Y, Zhao F, Chang H, Lyu J (2019) Longitudinal study of the relationship between sleep duration and hypertension in Chinese adult residents (CHNS 2004–2011). Sleep Med 58:88–92 10.1016/j.sleep.2019.01.006 31132577

[32] Lao XQ, Liu X, Deng HB, Chan TC, Ho KF, Wang F et al (2018) Sleep Quality, Sleep Duration, and the Risk of Coronary Heart Disease: A Prospective Cohort Study With 60,586 Adults. J Clin Sleep Med 14(1):109–117 10.5664/jcsm.6894 PMC5734879 29198294

[33] Nutt D, Wilson S, Paterson L (2008) Sleep disorders as core symptoms of depression. Dialogues Clin Neurosci 10(3):329–336 10.31887/DCNS.2008.10.3/dnutt PMC3181883 18979946

[34] Redeker NS, Jeon S, Muench U, Campbell D, Walsleben J, Rapoport DM (2010) Insomnia symptoms and daytime function in stable heart failure. Sleep 33(9):1210–1216 10.1093/sleep/33.9.1210 PMC2938862 20857868

[35] Lou P, Qin Y, Zhang P, Chen P, Zhang L, Chang G et al (2015) Association of sleep quality and quality of life in type 2 diabetes mellitus: a cross-sectional study in China. Diabetes Res Clin Pract 107(1):69–76 10.1016/j.diabres.2014.09.060 25458325

[36] Baylan S, Griffiths S, Grant N, Broomfield NM, Evans JJ, Gardani M (2020) Incidence and prevalence of post-stroke insomnia: a systematic review and meta-analysis. Sleep Med Rev 49:101222 10.1016/j.smrv.2019.101222 31739180

[37] Agusti A, Hedner J, Marin JM, Barbé F, Cazzola M, Rennard S (2011) Night-time symptoms: a forgotten dimension of COPD. Eur Respir Rev 20(121):183–194 10.1183/09059180.00004311 PMC9584119 21881146

[38] Kim JY, Kim N, Seo PJ, Lee JW, Kim MS, Kim SE et al (2013) Association of sleep dysfunction and emotional status with gastroesophageal reflux disease in Korea. J Neurogastroenterol Motil 19(3):344–354 10.5056/jnm.2013.19.3.344 PMC3714413 23875102

[39] Mehrdad M, Azarian M, Sharafkhaneh A, Alavi A, Zare R, Rad AH et al (2021) Association between poor sleep quality and glycemic control in adult patients with diabetes referred to Endocrinology Clinic of Guilan: a cross-sectional study. Int J Endocrinol Metab 20(1):e118077 10.5812/ijem.118077 PMC8994826 35432555

[40] Mehrdad M, Azarian M, Sharafkhaneh A, Alavi A, Leili EK, Rad AH et al (2023) The association between OSA and glycemic control in diabetes. Int J Prev Med 14(1):26 10.4103/ijpvm.ijpvm_356_21 PMC10080574 37033275

[41] Azarian M, Ramezani A, Sharafkhaneh A, Maghsoudi A, Kryger M, Thomas RJ et al (2025) The association between all-cause mortality and obstructive sleep apnea in adults: a U-shaped curve. Ann Am Thorac Soc 22(4):581–590 10.1513/AnnalsATS.202407-755OC PMC12005042 39746198

[42] Maghsoudi A, Azarian M, Sharafkhaneh A, Jones MB, Nozari H, Kryger M et al (2024) Age modulates the predictive value of self-reported sleepiness for all-cause mortality risk: insights from a comprehensive national database of veterans. J Clin Sleep Med 20(11):1785–1792 10.5664/jcsm.11254 PMC11530978 38935061

[43] Jeon C, Yan D, Nakamura M, Sekhon S, Bhutani T, Berger T et al (2017) Frequency and Management of Sleep Disturbance in Adults with Atopic Dermatitis: A Systematic Review. Dermatol Ther (Heidelb) 7(3):349–364 10.1007/s13555-017-0192-3 PMC5574743 28707054

[44] Sroka-Tomaszewska J, Bulińska B, Wilkowska A, Nowicki RJ, Trzeciak M (2023) Dupilumab for the treatment of moderate and severe atopic dermatitis: real-life experience. Postepy Dermatol Alergol 40(6):747–752 10.5114/ada.2023.133818 PMC10809831 38282886

[45] Yosipovitch G, Mollanazar N, Ständer S, Kwatra SG, Kim BS, Laws E et al (2023) Dupilumab in patients with prurigo nodularis: two randomized, double-blind, placebo-controlled phase 3 trials. Nat Med 29(5):1180–1190 10.1038/s41591-023-02320-9 PMC10202800 37142763

[46] Bender BG, Leung SB, Leung DY (2003) Actigraphy assessment of sleep disturbance in patients with atopic dermatitis: an objective life quality measure. J Allergy Clin Immunol 111(3):598–602 10.1067/mai.2003.174 12642843

[47] Kong TS, Han TY, Lee JH, Son SJ (2016) Correlation between severity of atopic dermatitis and sleep quality in children and adults. Ann Dermatol 28(3):321–326 10.5021/ad.2016.28.3.321 PMC4884708 27274630

[48] Yano C, Saeki H, Ishiji T, Ishiuji Y, Sato J, Tofuku Y et al (2013) Impact of disease severity on sleep quality in Japanese patients with atopic dermatitis. J Dermatol Sci 72(2):195–197 10.1016/j.jdermsci.2013.06.010 23876306

[49] Mann C, Dreher M, Weeß HG, Staubach P (2020) Sleep disturbance in patients with urticaria and atopic dermatitis: an underestimated burden. Acta Derm Venereol 100(6):adv00073 10.2340/00015555-3416 PMC9128904 32016441

[50] Li JC, Fishbein A, Singam V, Patel KR, Zee PC, Attarian H et al (2018) Sleep Disturbance and Sleep-Related Impairment in Adults With Atopic Dermatitis: A Cross-sectional Study. Dermatitis 29(5):270–277 10.1097/DER.0000000000000401 PMC6169311 30234614

[51] Luca M, Musumeci ML, D’Agata E, Micali G (2020) Depression and sleep quality in psoriatic patients: impact of psoriasis severity. Int J Psychiatry Clin Pract 24(1):102–104 10.1080/13651501.2019.1659372 31852324

[52] Kaaz K, Szepietowski JC, Matusiak Ł (2019) Influence of itch and pain on sleep quality in atopic dermatitis and psoriasis. Acta Derm Venereol 99(2):175–180 10.2340/00015555-3065 30307027

[53] Alatas ET, Unal Y, Demir Pektas S, Kutlu G (2020) Obstructive sleep apnea syndrome in patients with chronic idiopathic urticaria. Dermatol Ther 33(6):e14060 10.1111/dth.14060 32705737

[54] Hoskin B, Ortiz B, Paknis B, Kavati A (2019) Humanistic burden of refractory and nonrefractory chronic idiopathic urticaria: a real-world study in the United States. Clin Ther 41(2):205–220 10.1016/j.clinthera.2018.12.004 30606639

[55] Gimenéz-Arnau AM, Spector S, Antonova E, Trzaskoma B, Rosén K, Omachi TA et al (2016) Improvement of sleep in patients with chronic idiopathic/spontaneous urticaria treated with omalizumab: results of three randomized, double-blind, placebo-controlled studies. Clin Transl Allergy 6:32 10.1186/s13601-016-0120-0 PMC4989527 27540466

[56] Ismail AMA, El Gressy NSSA, Hegazy MD, Ahmed OSM, Elfahl AMAH (2025) Effects of diaphragmatic breathing exercise on sleeping quality, cortisol, cardiovascular autonomic functions, depression, and fatigue: a randomized-controlled trial in women with systemic sclerosis. Reumatologia (Warsz) 63(2):89–96 10.5114/reum/200193 PMC12138990 40485947

[57] Cherrez-Ojeda I, Maurer M, Felix M, Bernstein JA, Ramon GD, Jardim Criado RF et al (2021) Chronic urticaria and obstructive sleep apnea: is there a significant association? World Allergy Organ J 14(8):100577 10.1016/j.waojou.2021.100577 PMC8387911 34471460

[58] Forjaz MJ, Ayala A, Caminoa M, Prior N, Pérez-Fernández E, Caballero T (2021) HAE-AS: A Specific Disease Activity Scale for Hereditary Angioedema With C1-Inhibitor Deficiency. J Investig Allergol Clin Immunol 31(3):246–252 10.18176/jiaci.0479 31932270

[59] Weller K, Donoso T, Magerl M, Aygören-Pürsün E, Staubach P, Martinez-Saguer I et al (2020) Validation of the Angioedema Control Test (AECT)-A Patient-Reported Outcome Instrument for Assessing Angioedema Control. J Allergy Clin Immunol Pract 8(6):2050-2057.e2054 10.1016/j.jaip.2020.02.038 32173507

[60] Weller K, Magerl M, Peveling-Oberhag A, Martus P, Staubach P, Maurer M (2016) The Angioedema Quality of Life Questionnaire (AE-QoL) - assessment of sensitivity to change and minimal clinically important difference. Allergy 71(8):1203–1209 10.1111/all.12900 27038109

[61] Chung F, Yegneswaran B, Liao P, Chung SA, Vairavanathan S, Islam S et al (2008) STOP questionnaire: a tool to screen patients for obstructive sleep apnea. Anesthesiology 108(5):812–821 10.1097/ALN.0b013e31816d83e4 18431116

[62] Chung F, Subramanyam R, Liao P, Sasaki E, Shapiro C, Sun Y (2012) High STOP-Bang score indicates a high probability of obstructive sleep apnoea. Br J Anaesth 108(5):768–775 10.1093/bja/aes022 PMC3325050 22401881

[63] Roth T, Zammit G, Kushida C, Doghramji K, Mathias SD, Wong JM et al (2002) A new questionnaire to detect sleep disorders. Sleep Med 3(2):99–108 10.1016/s1389-9457(01)00131-9 14592227

[64] Benítez I, Roure N, Pinilla L, Sapiña-Beltran E, Buysse DJ, Barbé F et al (2020) Validation of the Satisfaction, Alertness, Timing, Efficiency and Duration (SATED) Questionnaire for Sleep Health Measurement. Ann Am Thorac Soc 17(3):338–343 10.1513/AnnalsATS.201908-628OC 31899656

[65] Arslan S, Tarı Selçuk K, Kirbiyik KB, Şerbetçi N (2025) The relationship between caffeine consumption and self-reported sleep quality among healthcare professionals. Adv Integr Med 12(4):100553

[66] Lechat B, Naik G, Reynolds A, Aishah A, Scott H, Loffler KA et al (2022) Multinight Prevalence, Variability, and Diagnostic Misclassification of Obstructive Sleep Apnea. Am J Respir Crit Care Med 205(5):563–569 10.1164/rccm.202107-1761OC PMC8906484 34904935

[67] Zhang Y, Ren R, Lei F, Zhou J, Zhang J, Wing YK et al (2019) Worldwide and regional prevalence rates of co-occurrence of insomnia and insomnia symptoms with obstructive sleep apnea: A systematic review and meta-analysis. Sleep Med Rev 45:1–17 10.1016/j.smrv.2019.01.004 30844624

[68] Meira ECM, Salles C, Gozal D (2021) A Reappraisal on the Associations between Sleep-disordered Breathing, Insomnia, and Cardiometabolic Risk. Am J Respir Crit Care Med 203(12):1583–1584 10.1164/rccm.202102-0337LE PMC8483231 33740388

[69] Gupta MA, Knapp K (2014) Cardiovascular and psychiatric morbidity in obstructive sleep apnea (OSA) with insomnia (sleep apnea plus) versus obstructive sleep apnea without insomnia: a case-control study from a Nationally Representative US sample. PLoS ONE 9(3):e90021 10.1371/journal.pone.0090021 PMC3943798 24599301

[70] Parthasarathy S, Vasquez MM, Halonen M, Bootzin R, Quan SF, Martinez FD et al (2015) Persistent insomnia is associated with mortality risk. Am J Med 128(3):268-275.e262 10.1016/j.amjmed.2014.10.015 PMC4340773 25447616

[71] Arslan S, Bozkurt C, Arslan M, Bulut H (2023) Effects of adherence to the Mediterranean diet on fatigue and activities of daily living in geriatric individuals with COPD. Clin Nutr ESPEN 54:436–442 10.1016/j.clnesp.2023.02.019 36963891

[72] Arslan S (2023) Exploring the potential of ChatGPT in personalized obesity treatment. Ann Biomed Eng 51(9):1887–1888 10.1007/s10439-023-03227-9 37145177

[73] Ismail AMA (2026) Obstructive sleep apnea: it is time to focus on performing aerobic exercise that may improve erectile dysfunction associated with the disease. J Sex Ment Health 24:e03326001

[74] Ismail AMA, Gadallah NGM, Elfahl AMA (2025) Apnea-hypopnea index, blood pressure, daytime sleepiness, and sleeping quality in obstructive sleep apnea: randomized-controlled responses to Shitali respiratory training. Respir Med. 10.1016/j.rmed.2025.108608 41448283

[75] El-Hadidy HA, Ismail AMA, El Sayed SG, Ahmed AM, Elgohary OM (2025) Additive effect of free walking exercise on liver enzymes, fatigue severity, triglycerides, and sleeping quality in obstructive sleep apnea patients with non-alcoholic fatty liver: randomized controlled trial. Adv Rehabil 39(4):16–27

[76] Ismail A, Mousa NMA, Elgendy SKM, Al-Emrany AM, Saber OSA, Elhakk SMA et al (2025) Effect of lifestyle changes on liver enzymes, triglycerides, sex hormones, and daytime sleepiness in polycystic ovarian syndrome women with obstructive sleep apnea and fatty liver-a randomized controlled trial. Menopause Rev 24(2):94–101 10.5114/pm.2025.152991 PMC12327225 40777876

