# Supplementary material for: Prevalence and impact of sleep disorders in patients with hereditary angioedema: A multicenter cross-sectional study in Latin America
Source: Sleep Breath. 2026 Sep 25;30(5):270. doi: 10.1007/s11325-026-03815-8 (PMC13615112; doi:10.1007/s11325-026-03815-8)
Supplement: Supplementary file 1 [file 11325_2026_3815_MOESM1_ESM.docx]

**eTable 1. Sleep disorder frequency by HAE treatment type.**

| Sleep disorder  n (%) | No treatment | On Demand | Long-term prophylaxis | Both | p |
| --- | --- | --- | --- | --- | --- |
| Insomnia | 14 (51.9%) | 31 (55.4%) | 3 (33.3%) | 27 (57.4%) | 0.60 |
| Hypersomnolence | 10 (37.0%) | 11 (19.6%) | 1 (11.1%) | 9 (19.1%) | 0.21 |
| OSA | 4 (14.8%) | 14 (25.0%) | 2 (22.2%) | 19 (40.4%) | 0.107 |
| SWSD | 3 (11.1%) | 11 (19.6%) | 1 (11.1%) | 9 (19.1%) | 0.73 |
| RLS/PMLD | 4 (14.8%) | 11 (19.6%) | 0 | 6 (12.8%) | 0.44 |
| REM-SBD | 2 (7.4%) | 4 (7.1%) | 0 | 1 (2.1%) | 0.54 |
| Parasomnia | 2 (7.4%) | 4 (7.1%) | 0 | 1 (2.1%) | 0.54 |
| SATED |  |  |  |  |  |
| Poor | 14 (51.9%) | 35 (62.5%) | 5 (55.6%) | 23 (48.9%) | 0.57 |
| Intermediate | 11 (40.7%) | 16 (28.6%) | 4 (44.4%) | 22 (46.8%) |  |
| Good sleep health | 2 (7.4%) | 5 (8.9%) | 0 | 2 (4.3%) |  |
| STOP-Bang |  |  |  |  |  |
| Low risk | 19 (70.4%) | 42 (75.0%) | 6 (66.7%) | 24 (51.1%) | 0.080 |
| Moderate-to-high risk | 8 (29.6%) | 14 (25.0%) | 3 (33.3%) | 23 (48.9%) |  |

HAE=hereditary angioedema; OSA=obstructive sleep apnea; REM-SBD=rapid eye movement sleep behavior disorder; RLS/PLMD=restless legs syndrome/periodic limb movement disorder; SATED=Satisfaction, Alertness, Timing, Efficiency, and Duration; STOP-Bang=Snoring, Tiredness, Observed apnea, high blood pressure, body mass index, age, neck circumference, and gender; SWSD=shift work sleep disorder.

Sleep disorder frequencies did not differ significantly by HAE treatment type. Insomnia, GSAQ-defined sleep apnea symptoms, SWSD, and RLS/PLMD were more frequent among participants receiving on-demand treatment or combined on-demand treatment and long-term prophylaxis, but these differences were descriptive.

**eTable 2. Sleep disorders by disease activity assessed by Hereditary Angioedema Activity Score (HAE-AS).**

| Sleep disorder  n (%) | HAE, Low activity, n (%) | HAE, High activity, n (%) | p |
| --- | --- | --- | --- |
| Insomnia | 24 (39.3%) | 51 (65.4%) | 0.002 |
| Hypersomnolence | 15 (24.6%) | 16 (20.5%) | 0.57 |
| GSAQ-defined sleep apnea symptoms | 12 (19.7%) | 27 (34.6%) | 0.050 |
| SWSD | 10 (16.4%) | 14 (17.9%) | 0.81 |
| RLS/PMLD | 3 (4.9%) | 18 (23.1%) | 0.003 |
| REM-SBD | 2 (3.3%) | 5 (6.4%) | 0.40 |
| Parasomnia | 2 (3.3%) | 5 (6.4%) | 0.40 |
| SATED |  |  |  |
| Poor | 29 (47.5%) | 48 (61.5%) | 0.23 |
| Intermediate | 28 (45.9%) | 25 (32.1%) |  |
| Good sleep health | 4 (6.6%) | 5 (6.4%) |  |
| STOP-Bang |  |  |  |
| Low risk | 45 (73.8%) | 46 (59.0%) | 0.070 |
| Moderate-to-high risk | 16 (26.2%) | 32 (41.0%) |  |

Exact P values are shown descriptively. OSA=obstructive sleep apnea; REM-SBD=rapid eye movement sleep behavior disorder; RLS/PLMD=restless legs syndrome/periodic limb movement disorder; SATED=Satisfaction, Alertness, Timing, Efficiency, and Duration; STOP-Bang=Snoring, Tiredness, Observed apnea, high blood pressure, body mass index, age, neck circumference, and gender; SWSD=shift work sleep disorder.

Patients with severe HAE activity had higher frequencies of insomnia (65.4% vs. 39.3%, P=0.002) and RLS/PLMD (23.1% vs. 4.9%, P=0.003) than patients with non-severe activity. GSAQ-defined sleep apnea symptoms were also more frequent in patients with severe activity (34.6% vs. 19.7%, P=0.050). STOP-Bang moderate-to-high OSA risk was 41.0% in patients with severe activity and 26.2% in patients with non-severe activity (P=0.070).

**eTable 3. Quality-of-life impairment by insomnia category.**

| AE-QoL domains  n (%) | No  64 (46.0%) | Yes  75 (54.0%) | Total  (n=139) | p-value |
| --- | --- | --- | --- | --- |
| Functioning | 43.066 (21.797) | 50.167 (25.397) | 46.897 (23.987) | 0.08 |
| Fatigue/Mood | 35.781 (16.360) | 64.933 (21.141) | 51.511 (23.969) | **<0.001** |
| Fears/shame | 48.633 (21.759) | 67.333 (24.755) | 58.723 (25.145) | **<0.001** |
| Nutrition | 39.453 (26.279) | 43.167 (31.185) | 41.457 (28.986) | 0.45 |

AE-QoL=angioedema quality of life

Participants with insomnia presented a higher mean percentage (SD) of QoL impairment as follows: fatigue/mood, 64.93 (21.14) vs 35.78 (16.360; and, fears/shame, 67.33 (24.76) vs 48.63 (21.76). The domains of functioning and nutrition did not show significant variation in the mean percentage of QoL impairment according to insomnia in patients with HAE.

**eTable 4. Quality-of-life impairment by OSA categories.**

| OSA Risk PerSTOP-BangCategory | | | | |
| --- | --- | --- | --- | --- |
| AE-QoL domains  n (%) | **No**  **91 (65.5%)** | **Yes**  **48 (34.5%)** | **Total**  **(n=139)** | **p-value** |
| Functioning | 44.5 (23.1) | 51.4 (25.20) | 46.897 (24.0) | 0.10 |
| Fatigue/Mood | 44.9 (22.6) | 63.958 (21.6) | 51.511 (24.0) | **<0.001** |
| Fears/shame | 57.509 (24.3) | 61.024 (26.7) | 58.723 (25.1) | 0.45 |
| Nutrition | 37.363 (29.0) | 49.219 (27.7) | 41.457 (29.0) | **0.02** |

AE-QoL=angioedema quality of life; OSA=obstructive sleep apnea; QoL=quality of life; STOP-Bang=Snoring, Tiredness, Observed apnea, high blood pressure, body mass index, age, neck circumference, and gender.

Participants with moderate-to-high risk of OSA presented a higher mean percentage (SD) of quality-of-life impairment as follows: fatigue/mood, 63.96 (21.63) vs. 44.95 (22.59); and nutrition, 49.22 (27.70) vs. 37.36 (28.96). The domains of functioning and fears/shame did not show significant variation in the mean percentage of QoL impairment according to STOP-Bang OSA risk in patients with HAE.
